# Converting Poly(Methyl Methacrylate) into a Triple‐Responsive Polymer

**DOI:** 10.1002/chem.202000485

**Published:** 2020-04-24

**Authors:** Christian Hils, Emma Fuchs, Franziska Eger, Judith Schöbel, Holger Schmalz

**Affiliations:** ^1^ Macromolecular Chemistry II Universität Bayreuth Universitätsstrasse 30 95440 Bayreuth Germany; ^2^ Macromolecular Chemistry & New Polymeric Materials Zernike Institute for Advanced Materials University of Groningen Nijenborgh 4 9747 AG Groningen Germany; ^3^ Keylab Synthesis and Molecular Characterization Bavarian Polymer Institute Universität Bayreuth Universitätsstrasse 30 95440 Bayreuth Germany

**Keywords:** pH-responsive polymers, polymer analogous modification, switchable surface hydrophilicity, temperature-responsive polymers

## Abstract

Multiresponsive polymers that can respond to several external stimuli are promising materials for a manifold of applications. Herein, a facile method for the synthesis of triple‐responsive (pH, temperature, CO_2_) poly(*N*,*N*‐diethylaminoethyl methacrylamide) by a post‐polymerization amidation of poly(methyl methacrylate) (PMMA) is presented. Combined with trivalent counterions ([Fe(CN)_6_]^3−^) both an upper and lower critical solution temperature (UCST/LCST)‐type phase behavior can be realized at pH 8 and 9. PMMA and PMMA‐based block copolymers are readily accessible by living anionic and controlled radical polymerization techniques, which opens access to various responsive polymer architectures based on the developed functionalization method. This method can also be applied on melt‐processed bulk PMMA samples to introduce functional, responsive moieties at the PMMA surface.

## Graphical abstract

**Making PMMA responsive**: PMMA can be converted into a triple‐responsive polymer, being responsive to temperature, pH and CO_2_ gas, by a quantitative post‐polymerization amidation with *N*,*N*‐diethylethylenediamine. This will open access to a large variety of responsive macromolecular architectures, because this method can be applied to PMMA and PMMA‐based block copolymers, being easily accessible by living anionic or controlled radical polymerization (see scheme).

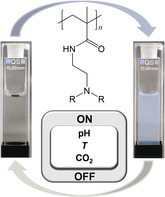

Stimuli‐responsive or “smart” polymers, which can change their physicochemical properties (e.g., solubility) upon applying an external stimulus (pH, temperature, light, magnetic fields, CO_2_, etc.), are highly attractive and intensively studied materials due to the wide range of applications, such as responsive micelles and micro/nano‐gels for biomedical applications, switchable membranes and coatings, smart actuators, or CO_2_ sensing.^1^, ^2^, ^3^ The most prominent examples of multi‐responsive polymers are based on methacrylate or acrylamide‐type monomers with pendant *N*,*N*‐dialkylamino groups (alkyl=methyl, ethyl, *iso*‐propyl; Scheme 1), which are commonly prepared by controlled radical polymerization techniques.^2^, ^3^, ^4^, ^5^, ^6^, ^7^, ^8^, ^9^ In contrast, there are considerably less reports on living anionic polymerization, for example, of *N*,*N*‐dimethylaminoethyl methacrylate (DMAEMA),^10^ despite the fact that anionic polymerization is still the best suited method to prepare well‐defined, complex block copolymer architectures of high molecular weight on a large scale.^11^ Especially, when soft blocks based on polydienes (polybutadiene, polyisoprene) are required to allow a dynamic rearrangement of micellar nanostructures or a later fixation of the structures by cross‐linking, anionic polymerization is the method of choice. However, the high requirements on monomer purity for anionic polymerization makes the purification of polar, high boiling monomers, such as DMAEMA, complex and time consuming. To overcome these limitations, we made use of an efficient post‐polymerization modification to convert poly(methyl methacrylate) (PMMA), which is easily accessible by living anionic, as well as controlled radical polymerization techniques, into a triple‐responsive polymer, being responsive to pH, temperature and CO_2_. This was realized by amidation of PMMA with different preactivated *N*,*N*‐dialkylethylenediamines to give the corresponding poly(*N*,*N*‐dialkylaminoethyl methacrylamide)s (alkyl=methyl, ethyl, *iso*‐propyl; PDxAEMAm), which were studied with respect to their responsive solution behavior. This concept was also applied for the surface functionalization of a bulk PMMA sample.

**Scheme 1:**
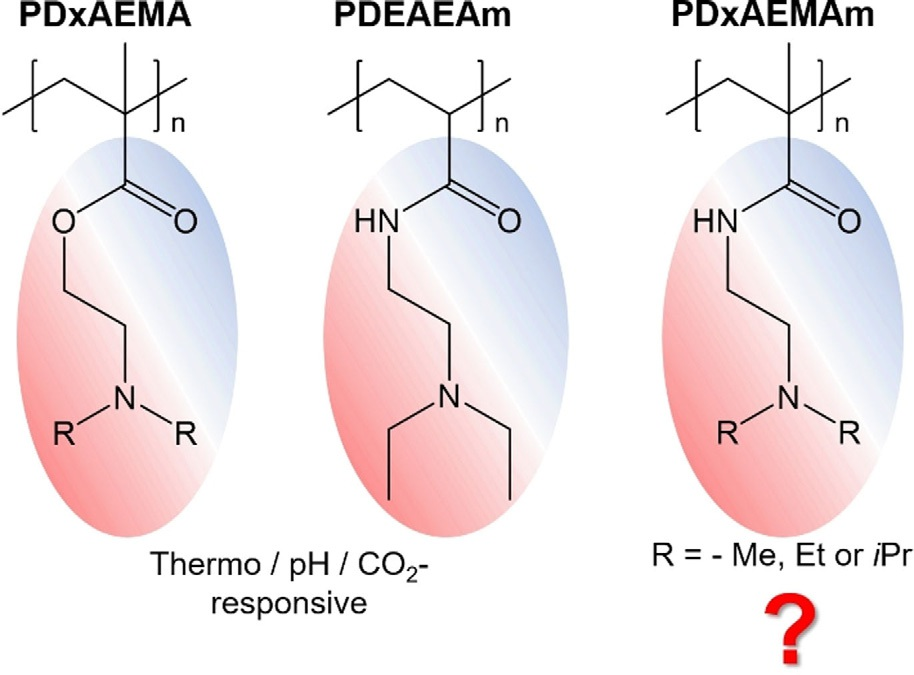
Comparison of the chemical structure of known triple‐responsive (pH, *T*, CO_2_) polymers with poly(*N*,*N*‐dialkylaminoethyl methacrylamide)s investigated in this study.

The amidation of PMMA with *N*,*N*‐dialkylethylenediamines was conducted according to our previously published method for the post‐polymerization functionalization of polystyrene‐*block*‐polyethylene‐*block*‐poly(methyl methacrylate) triblock terpolymers (Figure 1 A; details on used materials and synthesis protocols are given in the Supporting Information).^12^ Due to the prior activation of the amines with *n‐*butyllithium, quantitative functionalization can be reached in less than one hour, irrespective of the steric demand of the used amine (Figures S1, S2, and Table S1 in the Supporting Information), as was verified by ^1^H NMR and FTIR studies. In addition, there are no signs of a broadening of the molecular weight distribution by size‐exclusion chromatography (Figure S3), showing that amidation proceeds without significant side‐reactions. A quantitative conversion of the methyl ester groups of PMMA is indispensable to avoid hydrolysis to methacrylic acid at elevated temperatures and high pH values, which will cause a significant shift of the cloud point to higher temperatures with time.^8^ This is manifested by the disappearance of the lower critical solution temperature (LCST)‐type phase behavior of an intentionally prepared poly(methyl methacrylate‐*co*‐*N*,*N*‐diethylaminoethyl methacrylamide) copolymer (P(MMA_31_‐*co*‐DEAEMAm_179_), subscripts correspond to the degree of polymerization) already after nine consecutive heating/cooling cycles in pH 10 buffer solution (Figure S4 in the Supporting Information).

**Figure 1:**
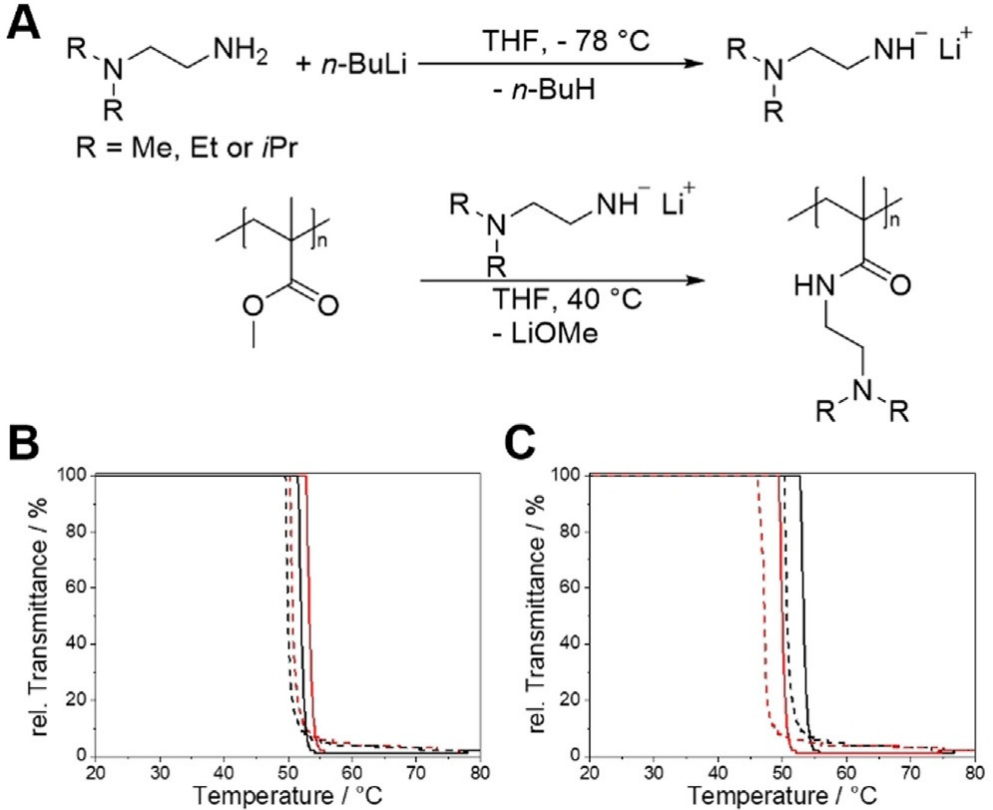
A) Reaction scheme for the amidation of PMMA with *N*,*N*‐dialkylethylenediamines. B) Temperature‐dependent transmittance of PDEAEMAm_210_ (*M*_n_=3.9×10^4^ g mol^−1^, 1st cycle: red, 9th cycle: black) and C) comparison with PDEAEMAm_1030_ (*M*_n_=1.9×10^5^ g mol^−1^, red trace) in pH 9 buffer. Heating traces are depicted as solid and cooling traces as dashed lines, respectively (*c=*1 g L^−1^).

The synthesized poly(*N*,*N*‐dimethylaminoethyl methacrylamide) (PDMAEMAm_210_) is neither responsive to pH nor to temperature, as was confirmed by turbidity measurements at varying pH (Figure S5 A in the Supporting Information). In contrast, poly(*N*,*N*‐diethylaminoethyl methacrylamide) (PDEAEMAm_210_, Figure 1 B) and poly(*N*,*N*‐di‐*iso*‐propylaminoethyl methacrylamide) (PD*i*PAEMAm_210_, Figure S5 B) exhibit a LCST‐type phase behavior at pH 8. However, only PDEAEMAm_210_ shows a narrow hysteresis (Δ*T*_CP_≈3 K), whereas for PD*i*PAEMAm_210_ the phase transitions upon heating and cooling are comparably broad with a large hysteresis (Δ*T*_CP_≈24 K). The cloud point (*T*_CP_) of PDEAEMAm_210_ changes only marginally after nine consecutive heating/cooling cycles in pH 9 buffer solution (1st cycle: *T*_CP_=53 °C, 9th cycle: *T*_CP_=52 °C, Figure 1 B), revealing the excellent hydrolytic stability of PDEAEMAm_210_. There is a concentration dependence of the cloud point, which leads to a pronounced shift of *T*_CP_ by approximately 20 °C to lower values with increasing concentration (*c=*0.05–2 g L^−1^, Figure S5 C in the Supporting Information). This is expected, because one moves along the binodal, which has a minimum in the LCST.

Figure 1 C reveals an influence of the molecular weight on the cloud point, because the *T*_CP_ of PDEAEMAm_1030_ is about 5 °C lower compared to that of PDEAEMAm_210_. This indicates that PDEAEMAm acts as an LCST polymer of class I, that is, the cloud point decreases with increasing molecular weight.^13^

Turbidity measurements were conducted in buffer solutions of different pH (Figure 2 A and Table S2 in the Supporting Information) to further study the potential multiresponsivity of PDEAEMAm. PDEAEMAm is soluble over the entire temperature range for pH≤7, whereas for 8<pH<10, the cloud point shifts from *T*_CP_=72 °C at pH 8 to *T*_CP_=37 °C at pH 10. This matches well with the measured p*K*_a_ value of 7.1 (Figure S6 in the Supporting Information), that is, an LCST‐type phase behavior is only observed for pH values at which less than 50 % of the pendant tertiary amino groups are protonated. This is consistent with studies on the chemically similar poly(*N*,*N*‐diethylaminoethyl methacrylate).^4^, ^9^ The pH dependence of the cloud point can be harnessed for a reversible, CO_2_‐induced phase transition (Figure 2 B). Bubbling CO_2_ through a turbid solution of PDEAEMAm_210_ in pH 10 buffer at 55 °C, that is, above the *T*_CP_ of 37 °C at pH 10, results in a complete dissolution of the polymer. This is caused by a decrease in solution pH by the dissolved CO_2_ (chemical equilibrium with carbonic acid) and consequently by the protonation of the pendant diethylamino groups as was proven by ^1^H NMR analysis (Figure S7 in the Supporting Information). Subsequent bubbling of nitrogen or argon to remove the dissolved CO_2_ gave again a turbid solution. This process can be repeated several times, proving the reversibility of the CO_2_‐induced solubility switching.

**Figure 2:**
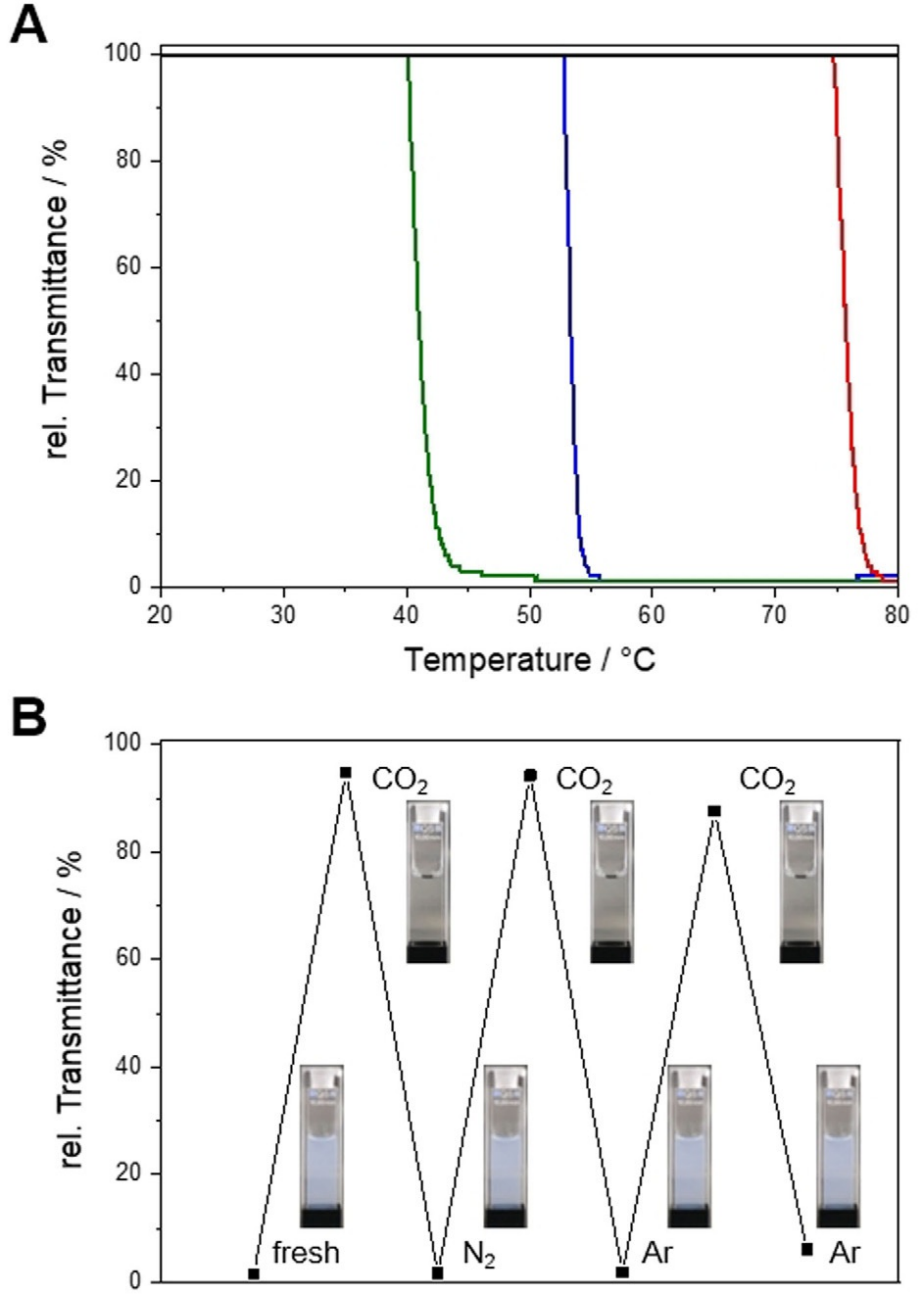
A) Temperature‐dependent transmittance of PDEAEMAm_210_ in buffer solutions of different pH (*c=*1 g L^−1^, pH 7 black, pH 8 red, pH 9 blue and pH 10 green). B) Change in transmittance of PDEAEMAm_210_ in pH 10 buffer at 55 °C (*c=*1 g L^−1^) upon bubbling CO_2_, N_2_ or Ar through a cuvette.

In comparison to the respective methacrylate‐based poly(*N*,*N*‐dialkylaminoethyl methacrylate)s, the replacement of the ester linkage by an amide linkage in poly(*N*,*N*‐dialkylaminoethyl methacrylamide)s leads to an increase in polarity and, thus, to an increased solubility. This is manifested by the fact that poly(*N*,*N*‐dimethylaminoethyl methacrylate) (PDMAEMA) shows a pH‐dependent LCST‐type phase behavior for pH≥7,^4^, ^8^ whereas PDMAEMAm is completely soluble irrespective of temperature and pH (Figure S5 A; Tables S2 and S3 in the Supporting Information). A similar behavior is found for the diethyl derivatives. PDEAEMA shows an LCST‐type phase behavior at pH 6–7 and is hardly soluble for pH≥8.^9^ In contrast, PDEAEMAm exhibits a temperature‐dependent solubility for pH≥8. This is in line with the lower p*K*_a_ value observed for PDEAEMA (p*K*_a_=6.6)^9^ with respect to that of PDEAEMAm (p*K*_a_=7.1, Figure S6 in the Supporting Information). In analogy to poly(*N*,*N*‐diethylaminoethyl acrylamide) (PDEAEAm) the synthesized PDEAEMAm also shows a triple‐responsive behavior, being responsive to pH, temperature and CO_2_.^5^, ^7^ However, introducing a methyl group in α‐position leads to a slightly increased hydrophobicity and a resulting shift of the critical pH, at which an LCST‐type phase behavior was observed, from pH 8.5 for PDEAEAm to pH 8 for PDEAEMAm, respectively (Table S2 in the Supporting Information).

In addition to the pH‐dependent LCST‐type phase behavior an upper critical solution temperature (UCST)‐type phase behavior can be induced by the addition of small quantities of a trivalent counterion. This is realized by the addition of K_3_[Fe(CN)_6_] to the respective solutions of PDEAEMAm_1030_ (*c=*1 g L^−1^) in buffers of pH 6–10 (Figure 3 A and Figure S8 in the Supporting Information). For pH 8 and 9, both an UCST‐ and LCST‐type phase behavior was observed, whereas for pH<8 and pH>9, only an UCST or LCST behavior can be detected, respectively. This can be explained by the lack of protonated (charged) repeating units for pH>9, as electrostatic interactions between the positively charged polymer and the trivalent [Fe(CN)_6_]^3−^ counterions are responsible for the UCST‐type phase behavior.^14^ Consequently, at pH<8 the polymer chain is highly charged (p*K*_a_=7.1), which leads to a vanishing of the LCST. In analogy to studies on linear and star‐shaped PDMAEMA, the UCST‐type cloud point increases with the [Fe(CN)_6_]^3−^ concentration, whereas the LCST‐type cloud point is not affected (Figure 3 B). However, the UCST‐type phase transitions for PDEAEMAm_1030_ are more sensitive to the [Fe(CN)_6_]^3−^ concentration and the UCST coincides with the LCST‐type cloud point already at *c*([Fe(CN)_6_]^3−^)=1.25 mm.

**Figure 3:**
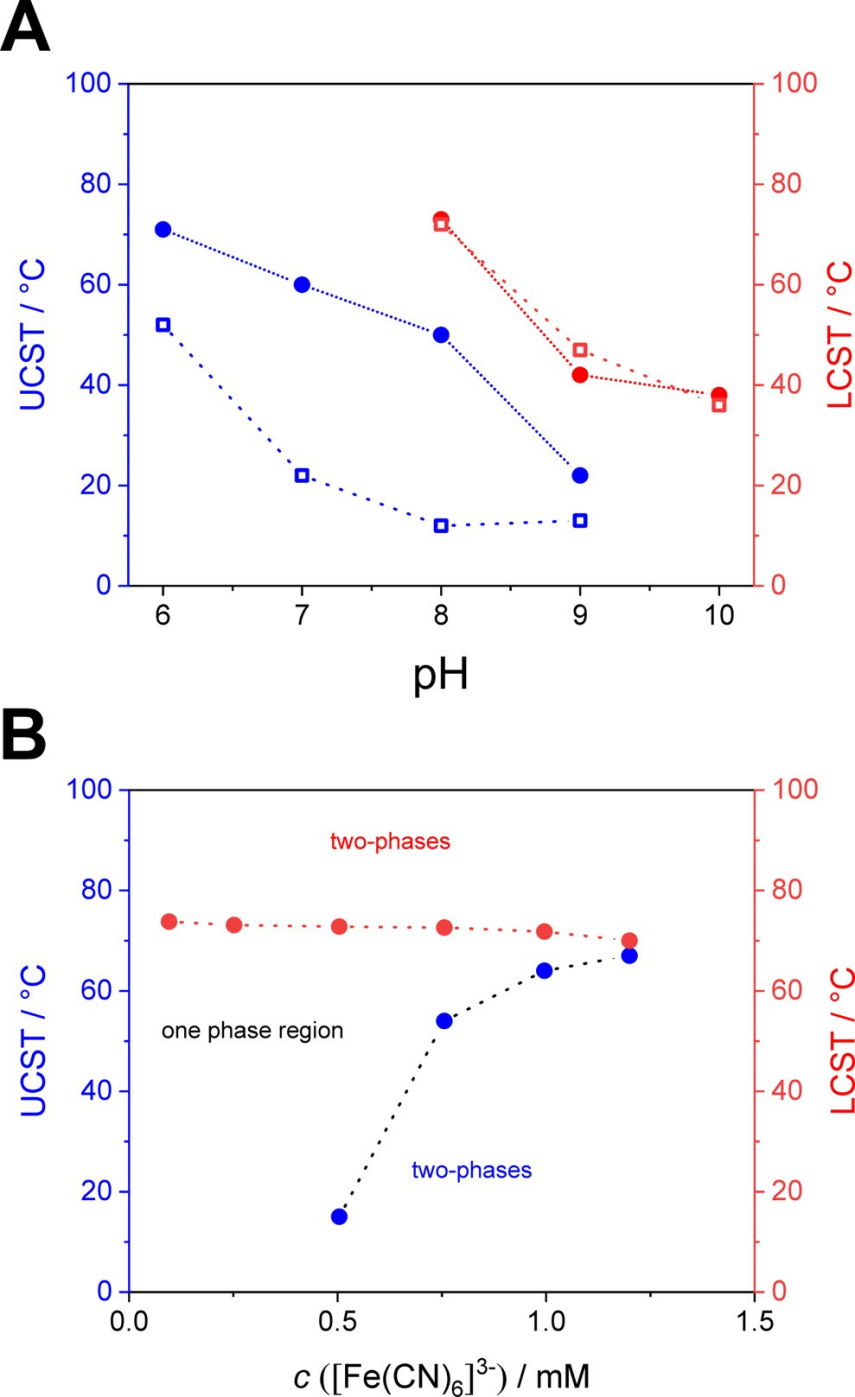
Tailoring the thermo‐responsive solution behavior of PDEAEMAm_1030_ (*c=*1 g L^−1^) in the presence of trivalent [Fe(CN)_6_]^3−^ counterions. A) UCST‐ and LCST‐type phase transitions in dependence of pH for two different K_3_[Fe(CN)_6_] concentrations (*c*=0.5 mm (squares), *c*=0.75 mm (circles)) and B) in dependence of K_3_[Fe(CN)_6_] concentration in pH 8 buffer solutions.

The post‐polymerization amidation of PMMA can even be conducted in bulk, allowing the direct heterogeneous amidation of melt‐processed PMMA parts. The successful amidation of the surface of a PMMA disc with *N*,*N*‐diethylethylenediamine was proven by FTIR spectroscopy, revealing the presence of the characteristic amide band at ≈1650 cm^−1^ (Figure S9 in the Supporting Information). Due to the increase in polarity the contact angle to water at 25 °C decreases from (93±2)° to (49±5)° after amidation (Table S4 in the Supporting Information). The responsivity of the amidated PMMA surface can be used for a temperature‐induced switching of the contact angle. Employing a pH 10 buffer solution a shift of the contact angle from (48±6)° to (77±1)° can be induced by a temperature increase to 55 °C, because under these conditions, the PDEAEMAm units become insoluble (*T*_CP_=37 °C at pH 10). Moreover, the diethylamino anchor groups at the PMMA surface can be utilized to bind preformed, citrate‐stabilized gold nanoparticles (Au NPs, *D*=9.5±2.4 nm). After functionalization and loading with Au NPs the decoration of the PMMA surface with Au NPs is clearly visible in the digital photograph, as well as the scanning electron microscopy (SEM) image acquired with a back‐scattered electron (BSE) detector (Figure 4).

**Figure 4:**
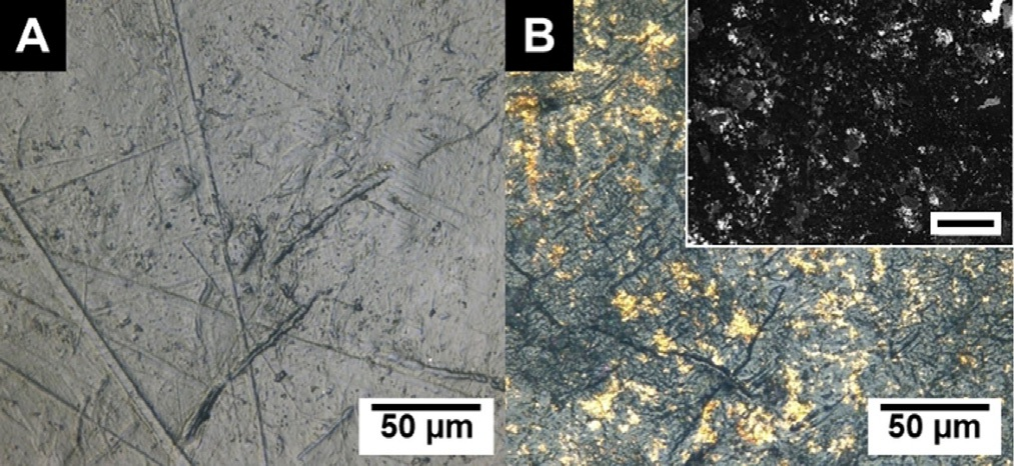
Digital photographs of the surface of the PMMA disc employed for heterogeneous amidation (A) and of the surface of the PMMA disc after amidation and successive loading with Au NPs (B). The inset shows the corresponding SEM image acquired with a BSE detector (Au NP rich regions appear bright, scale bar inset=100 μm).

In conclusion, we have shown that PMMA can be converted to a triple‐responsive (pH, temperature, CO_2_) polymer by a fast and quantitative post‐polymerization amidation with *N*,*N*‐diethylethylenediamine. This opens access to a variety of responsive polymer architectures, such as defined (multi)block copolymers,^12^ because PMMA is easily accessible by controlled radical, as well as living anionic polymerization. The excellent efficiency of this functionalization reaction also allows a direct heterogeneous amidation of the surface of melt‐processed PMMA parts, which can be harnessed for a temperature‐induced switching of the surface hydrophilicity or the binding of metal nanoparticles, for example, for catalytic purposes. Hence, we believe that the herein established method will find broad application in the synthesis of responsive and/or functional materials that might find application in responsive gels, actuators, or catalysis.

## Conflict of interest

The authors declare no conflict of interest.

## Supporting information

As a service to our authors and readers, this journal provides supporting information supplied by the authors. Such materials are peer reviewed and may be re‐organized for online delivery, but are not copy‐edited or typeset. Technical support issues arising from supporting information (other than missing files) should be addressed to the authors.

Supplementary
