# Supplementary material for: Converting Poly(Methyl Methacrylate) into a Triple‐Responsive Polymer
Source: Chemistry. 2020 Apr 24;26(25):5611–4. doi: 10.1002/chem.202000485 (PMC7317794; doi:10.1002/chem.202000485)
Supplement: Supplementary file 1 — Supplementary [file CHEM-26-5611-s001.pdf]

Chemistry–A European Journal

Supporting Information

Converting Poly(Methyl Methacrylate) into a Triple-Responsive Polymer

Christian Hils,[a] Emma Fuchs,[a] Franziska Eger,[a] Judith Schöbel,[b] and Holger Schmalz*[a, c]

Table of Contents

General Procedures

Synthesis Procedures

Synthesis of citrate-stabilized gold nanoparticles (Au NPs)

Synthesis of PDxAEMAm

Heterogeneous amidation and loading with Au NPs

Characterization

^{1}H-NMR

FT-IR

HFIP-SEC

Temperature-dependent transmittance

µDSC

Determination of pK_a

Temperature-dependent transmittance – switching the solubility behaviour with $K_3[Fe(CN)_6]$

Comparison of cloud points

Heterogeneous amidation

FT-IR

Contact angle

General Procedures

Materials. All chemicals were used as received unless otherwise specified. Deionized water (filtered through a Millipore Milli-Q Plus system, QPAK® 2, conductivity: 18.2 MΩ·cm), poly(methyl methacrylate) (PMMA, Acros Organics, M_w = 3.5·10^4 g·mol^{-1} ($PMMA_{210}$) and M_w = 1.2·10^5 g·mol^{-1} ($PMMA_{1030}$)), *n*-butyllithium (*n*-BuLi, 2.5M in hexane, Acros Organics), *n*-pentane (technical grade, purified by distillation prior to use), deuterated chloroform ($CDCl_3$, 99.8%, Deutero), calcium hydride (CaH_2, Merck), *N*,*N*-dimethylethylenediamine (DMEDA, ≥ 98%, Sigma-Aldrich), *N*,*N*-diethylethylenediamine (DEEDA, 99%, Acros Organics), *N*,*N*-di*iso*propylethylenediamine (D*i*PEDA, 97%, Acros Organics), dry ice, hydrochloric acid (37 wt% in water, VWR), tetrahydrofuran (THF, ≥ 99.9%, Sigma-Aldrich), acetone (99.9%, Acros Organics), buffer solutions (VWR, AVS Titrinorm (pH = 6 – 9) and Merck, Certipur (pH = 10)), potassium hexacyanoferrate(III) ($K_3[Fe(CN)_6]$, ReagentPlus®, ~99%, Sigma-Aldrich), diethyl ether (Et_2O, technical grade, purified by distillation prior to use), tetrachloroauric(III) acid trihydrate ($HAuCl_4 \cdot 3\,H_2O$, 99.99%, Alfa Aesar), sodium borohydride ($NaBH_4$, ≥ 96%, Fluka), and trisodium citrate dihydrate (99% for analytical purposes, Grüssing).

Purification of chemicals. The used PMMAs were dissolved in THF and precipitated from MeOH in order to obtain a fine powder ($PMMA_{210}$: M_n = 2.1·10^4 g·mol^{-1}, $Đ$ = 1.50; $PMMA_{1030}$: M_n = 1.0·10^5 g·mol^{-1}, $Đ$ = 1.17; SEC-MALLS combination; subscript denotes the degree of polymerization). *N*,*N*-dialkylethylenediamines were dried over CaH_2 and purified by distillation (stored under argon until use). THF was dried by successive distillation over CaH_2 and potassium (stored under nitrogen until use).

Nuclear Magnetic Resonance (NMR) spectroscopy. The polymers were characterized by ^{1}H-NMR spectroscopy (Bruker Ultrashield 300 spectrometer) using $CDCl_3$ as solvent. The signal assignment was supported by simulations with the NMR software *MestReNova*.

Fourier Transform-Infrared Spectroscopy (FT-IR). The FT-IR spectra were recorded with a Digilab Excalibur 3000 FT-IR instrument equipped with an ATR unit (diamond) at a resolution of 4 cm^{-1} (16 scans).

Size Exclusion Chromatography (SEC). HFIP-SEC was performed on an instrument equipped with three PSS-PFG gel columns (particle size = 7 µm) with porosity range from 100 – 300 Å (PSS, Mainz, Germany) together with a refractive index detector (Gynkotek). HFIP (hexafluoro*iso*propanol, HPLC grade, Appollo Scientific Limited) with potassium trifluoroacetate (98%, Sigma-Aldrich, 4.8 g in 600 mL HFIP) was used as eluant at a flow rate of 0.5 mL·min^{-1}, employing toluene (HPLC grade, Sigma-Aldrich) as internal standard. The calibration was done with narrowly distributed poly(methyl methacrylate) (PMMA) homopolymers (PSS calibration kit). The samples were dissolved in HFIP with potassium trifluoroacetate and filtered through a 0.22 µm PTFE filter before analysis. An injection volume of 20 µL was used for the measurements and the SEC columns were maintained at room temperature. The molar masses reported for the amidated PMMA samples are given in reference to PMMA standards.
The SEC-MALLS (THF) measurements for PMMA (d*n*/d*c* = 0.087 mL·g^{-1}) were performed on an instrument having three PSS-SDV gel columns (particle size = 5 µm) with porosity range from 10^3 to 10^5 Å (PSS, Mainz, Germany) together with a refractive index detector (Agilent, G1362A 1200 Series) as concentration detector and a multi-angle laser light scattering detector (MALLS) with eighteen different angles (Wyatt Technology, Dawn EOS). THF (99.9%, Fisher Scientific) was used as eluent at a flow rate of 0.8 mL·min^{-1}. The normalization and determination of the inter detector delay as well as the detector constants were done with a narrowly distributed polystyrene standard (PSS, Mainz, Germany). An injection volume of 20 µL was used and the measurements were carried out at room temperature.

Turbidity measurements. Freshly prepared polymer solutions (c = 1 g·L^{-1}) in the corresponding media were analysed with a Crystal16 multiple reactor system (Technobis Crystallization Systems, screw cap vials) or an UV-Vis spectrophotometer (JASCO V 630, wavelength accuracy: ± 0.7 nm; quartz glass cuvettes (D = 10 mm); halogen lamp as light source and a JASCO ETCS 761 sample holder) at a wavelength of λ = 645 nm and a scanning rate of 1.0 K·min^{-1}. The cloud points (T_{CP}) were calculated from the intersection of the tangents at the onset of turbidity.

Scanning Electron Microscopy (SEM). The SEM measurements were performed on a Zeiss 1530 with field emission cathode at an acceleration voltage of 10 kV using a back-scattering electron (BSE) detector. The samples were applied to a conductive adhesive carrier mounted on an aluminium plate and fixed with an aluminium adhesive strip.

Optical Microscope (OM). The measurements were performed on a Keyence VHX-100.

Contact Angle (CA) measurements. The CA measurements were performed employing a DS25 from Krüss. The CA was determined with the software "*Advanced Drop Shape Analysis*" (version 1.3.1.0) and the method "*Ellipse Tangent Sessile Drop*" using a droplet volume of V = 5 µL (deionized water or pH = 10 buffer solution). For the measurements at 55 °C a Mettler FP82 hot stage (FP80 Central Processor) was used.

Synthesis Procedures

Synthesis of citrate-stabilized gold nanoparticles (Au NPs). Citrate-stabilized Au NPs were prepared according to the method reported by *Schaal et al.*[1] To 100 mL of an aqueous $HAuCl_4$ solution (c = 0.50·10^{-3} mol·L^{-1}) 9 mL of a $NaBH_4$ solution in water (c = 4.85·10^{-2} mol·L^{-1}) were added dropwise followed by stirring for 5 min. The reaction mixture changed colour from yellow to red, indicating the formation of Au NPs. Subsequently, 5 mL of an aqueous sodium citrate solution (c = 0.05 mol·L^{-1}) were added and the mixture was stirred for another 5 min. Au NPs with a mean diameter of D_{TEM} = 9.5 ± 2.4 nm, as determined by transmission electron microscopy (TEM), were obtained.

Synthesis of PDxAEMAm. The post-polymerization amidation of PMMA with the corresponding *N*,*N*-dialkylethylenediamines (DMEDA, DEEDA and D*i*PEDA) was conducted under an inert argon atmosphere.[2] The amines were dissolved in THF (c = 0.25 – 1 mol·L^{-1}) and the solution was cooled to -78 °C (acetone/dry ice) before *n*-BuLi (1 eq. with respect to amine) was added. After 30 min the cooling bath was removed and the activated amines were transferred to a solution of PMMA in THF (c = 10 g·L^{-1}), followed by heating to 40 °C and stirring for 4 h. The reaction was quenched with 2 mL of deionized water. After precipitation from *n*-pentane, the polymers were filtered and dried in vacuum. The amidation resulted in quantitative functionalization of PMMA, as confirmed by ^{1}H-NMR and FT-IR spectroscopy (Figure S1, S2). The following poly(*N*,*N*-dialkylaminoethyl methacrylamide)s (PDxAEMAm) were prepared: $PDMAEMAm_{210}$, P(MMA_{31}-*co*-$DEAEMAm_{179}$), $PDEAEMAm_{210}$, $PDEAEMAm_{1030}$, and PD*i*$PAEMAm_{210}$ (subscripts correspond to the degree of polymerization).

Heterogeneous amidation and loading with Au NPs. First, the PMMA disc (Plexiglas® 7N, Röhm GmbH: M_n = 5.9·10^4 g·mol^{-1}, $Đ$ = 1.67; 9 x 8 x 1 mm^3, 0.13 g, 1 eq.) was washed with Et_2O and deionized water and dried in vacuo. *N*,*N*-Diethylethylenediamine (360 µL, 2 eq.) was dissolved in 10 mL Et_2O and cooled to -78 °C (acetone/dry ice) before *n*-BuLi (1.04 mL, 2 eq.) was added. After 10 min the PMMA disc was added followed by stirring for another 5 min. Subsequently, the yellowish reaction mixture was heated to 25 °C and stirred for 15 h. The reaction was quenched by adding 2 mL deionized water and the amidated PMMA disc was washed with deionized water and dried in vacuo.
For the loading with Au NPs, a piece of the amidated PMMA disc (7 x 4 x 1 mm^3) was added to a mixture of 1 mL deionized water and 0.5 mL of an aqueous, citrate-stabilized AuNP dispersion (c = 0.5·10^{-3} mol·L^{-1}) and shaken for 15 h at 25 °C (400 rpm, thermal shaker HLC MKR-13, Ditabis). Afterwards, the disc was washed with a sodium citrate solution (2 x 5 mL; c = 0.05 mol·L^{-1}) and deionized water (2 x 5 mL).

Characterization

Kinetics of the amidation of PMMA, as studied by ^{1}H-NMR

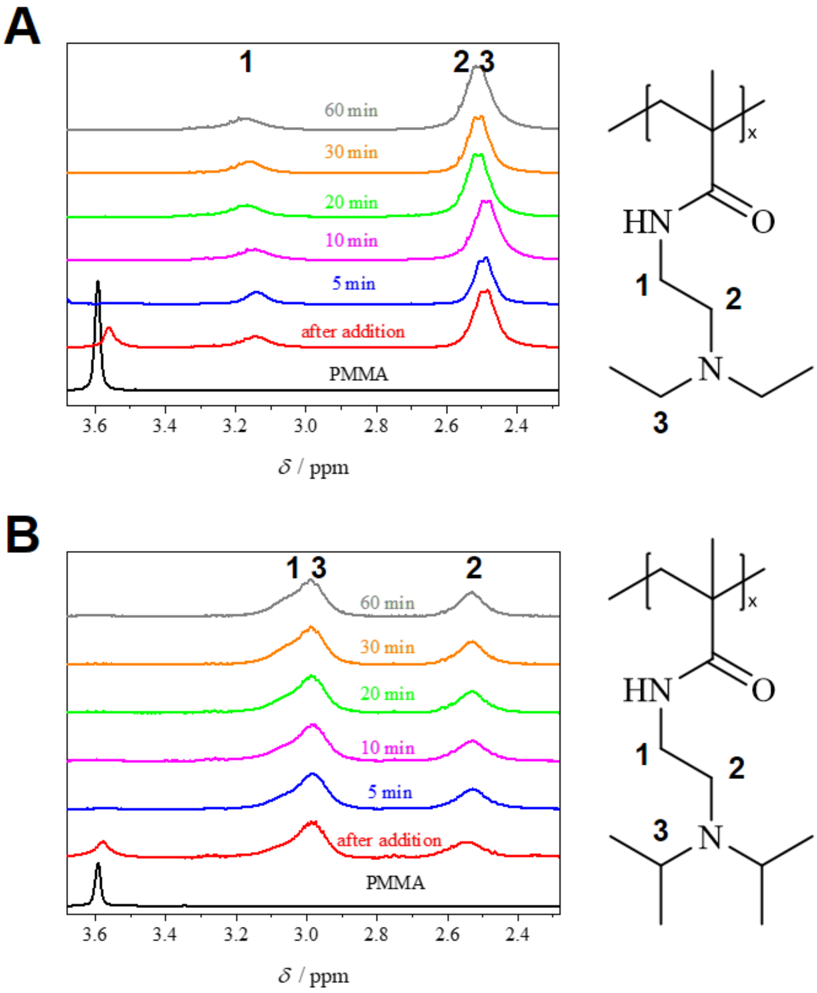

Figure S1. Kinetics of the amidation of PMMA with DEEDA (A) and D*i*PEDA (B) studied by ^{1}H-NMR in $CDCl_3$. The signal at δ = 3.6 ppm can be assigned to the methoxy protons of the ester units of PMMA.

Kinetics of the amidation of PMMA, as studied by FT-IR spectroscopy

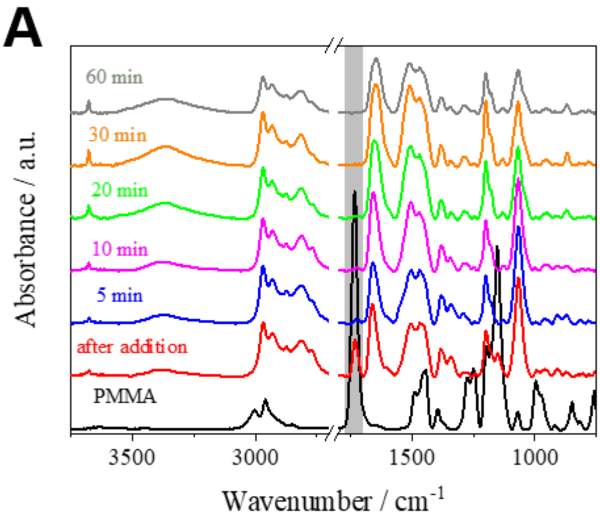

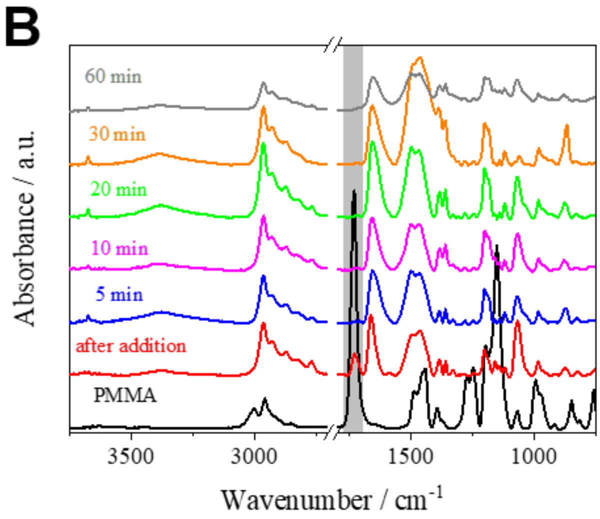

Figure S2. Kinetics of the amidation of PMMA with DEEDA (A) and D*i*PEDA (B) studied by FT-IR spectroscopy. The progress of the reaction can be monitored by the decrease of the PMMA ester band at $\nu \approx 1730$ cm^{-1} (marked in grey) and the corresponding appearance/increase of the amide band at $\nu \approx 1650$ cm^{-1}, respectively.

Overview of the synthesized polymers using $PMMA_{210}$ and $PMMA_{1030}$ as precursor

Table S1. Overview of the synthesized polymers using $PMMA_{210}$ and $PMMA_{1030}$ as precursor.

polymer	eq. amine	degree of amidation / % [a]	M_n / g·mol^{-1} [b]
$PDEAEAm_{210}$ [c]	2	≥ 99	39 000
$PDEAEAm_{1030}$ [c]	2	≥ 99	190 000
P(MMA_{31}-*co*-$DEAEAm_{179}$) [d]	1.7	85	36 000
PD*i*$PAEMAm_{210}$ [e]	2	≥ 99	44 600

[a] with respect to converted methyl ester units of PMMA (-O-CH_3, δ = 3.6 ppm), as determined by ^{1}H-NMR, [b] calculated using the degree of polymerization of the precursor PMMA (determined by SEC-MALLS) and the respective degree of amidation (determined by ^{1}H-NMR), [c] **p**oly(**die**thyl**a**mino**e**thyl **m**ethacryl**am**ide), [d] **p**oly(**m**ethyl **m**eth**a**crylate-*co*-**d**i**e**thyl**a**mino**e**thyl **m**ethacryl**am**ide), [e] **p**oly(**d**i*iso***p**ropyl**a**mino**e**thyl **m**ethacryl**am**ide).

HFIP-SEC

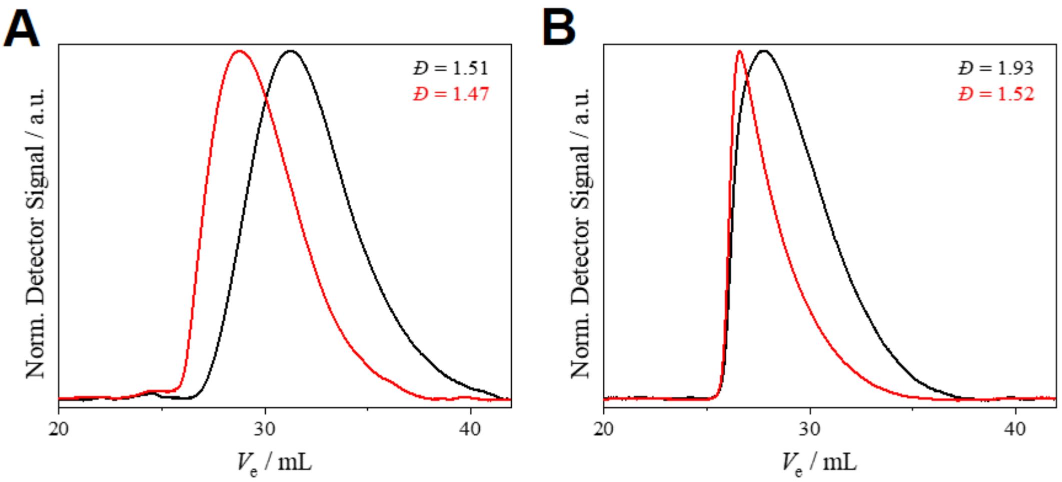

Figure S3. HFIP-SEC of A) $PMMA_{210}$ (black), $PDEAEMAm_{210}$ (red) and B) $PMMA_{1030}$ (black), $PDEAEMAm_{1030}$ (red). The asymmetric shape of the SEC trace of $PDEAEMAm_{1030}$ is due to the fact that the molecular weight is close to the molecular weight cut-off of the employed columns. Nevertheless, it shows that no by-products of lower molecular weight were formed during amidation.

Temperature-dependent transmittance

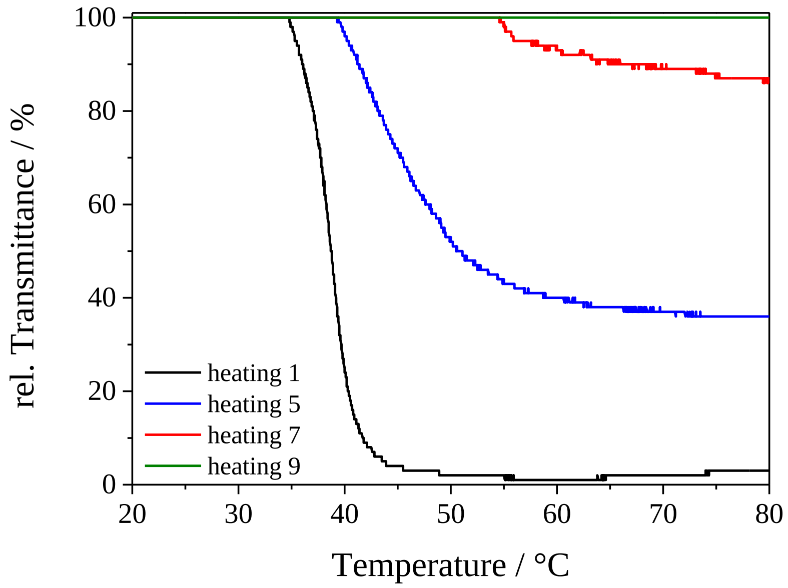

Figure S4. Temperature-dependent transmittance of P(MMA_{31}-*co*-$DEAEMAm_{179}$) in pH = 10 buffer solution (c = 1 g·L^{-1}).

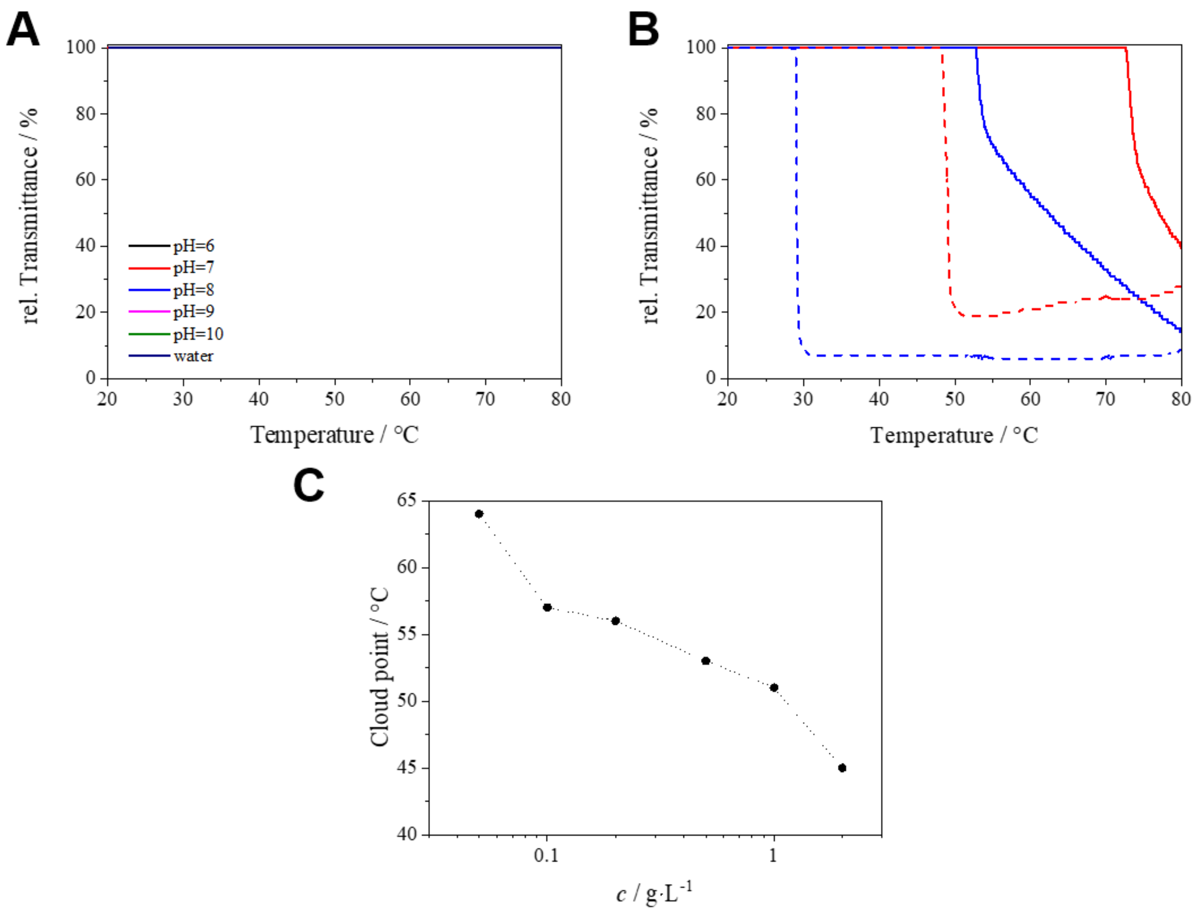

Figure S5. Temperature-dependent transmittance of A) PDMAEMAm$_{210}$ (c = 1 g·L^{-1}) in buffer solutions of different pH as indicated, B) PD*i*PAEMAm$_{210}$ (c = 1 g·L^{-1}) in pH = 7 (red traces) and pH = 8 (blue traces) buffer solutions (solid lines represent heating and dashed lines cooling traces, respectively) and C) concentration-dependent cloud points of PDEAEMAm$_{210}$ in pH = 9 buffer solution.

Determination of pK_a for PDEAEMAm$_{210}$

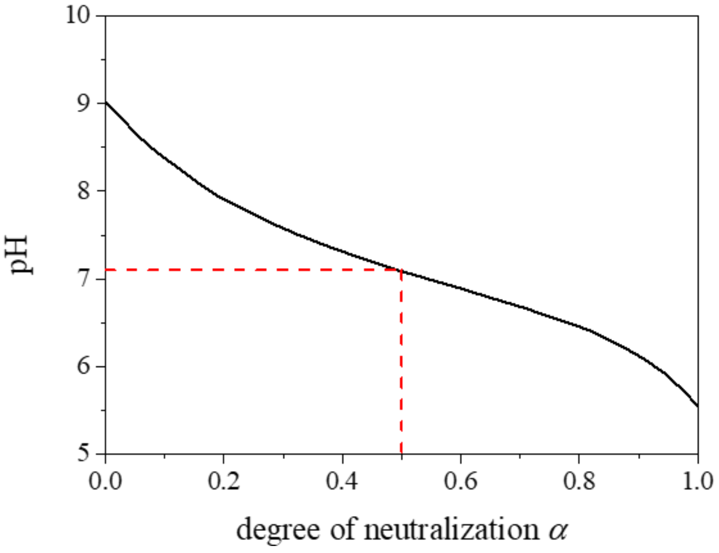

Figure S6. Titration curve of PDEAEMAm$_{210}$ (c = 1 g·L^{-1}) in deionized water with 0.1N HCl.

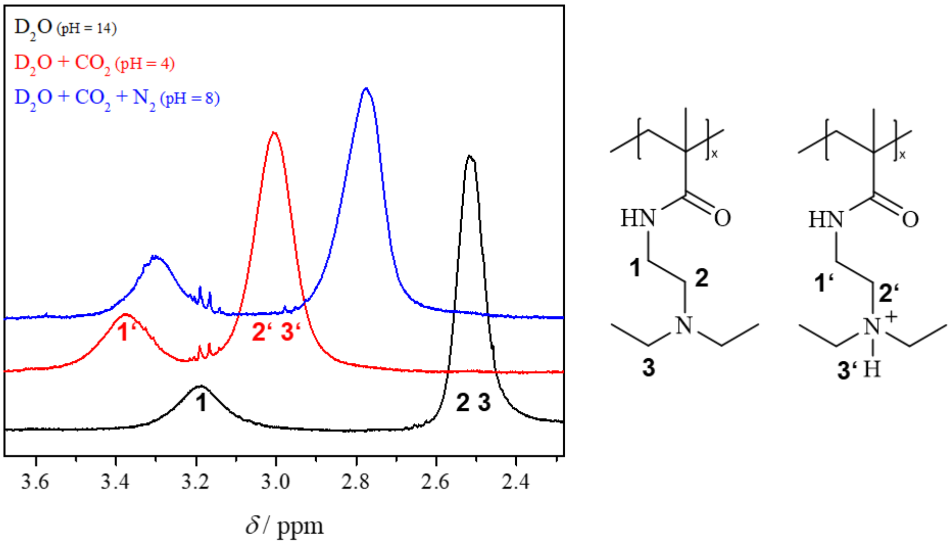

Figure S7. ^{1}H NMR spectra of PDEAEMAm$_{210}$ (c = 10 g·L^{-1}) in D_2O directly after dissolution (black), after bubbling of CO_2 through the solution (red) and after bubbling of nitrogen to remove most of the dissolved CO_2 (blue). Due to protonation of the pendant amino groups the respective signals are reversibly shifted downfield.

Comparison of cloud points

Table S2. Comparison of cloud points for (meth)acryl amide-based polymers.

polymer	c / g·L^{-1}	pH	T_{CP} [a]/ °C	Reference
PDMAEMAm$_{210}$	1	6-10	soluble	this work
PDEAEMAm$_{210}$	1	6	soluble	
	1	7	soluble	
	1	8	72	
	0.05	9	64	
	0.1	9	57	
	0.2	9	56	
	0.5	9	54	
	1	9	53	
	2	9	45	
	1	10	37	
PDEAEMAm$_{1030}$	1	9	49	
PD*i*PAEMAm$_{210}$	1	7	73	
	1	8	53	
PDMAEAm$_{144}$ [b]	1	14	soluble	3
PDEAEAm$_{130}$ [c]	0.5	8	soluble	
	0.5	8.5	50.2	
	0.5	11	33.1	
	0.5	13	31.5	
	0.5	14	27.5	

[a] λ = 645 nm for PDxAEMAm$_{210}$, λ = 500 nm for PDMAEAm$_{144}$ and PDEAEAm$_{130}$, [b] **p**oly(**dim**ethyl**a**mino**e**thyl acryl**am**ide), [c] **p**oly(**die**thyl**a**mino**e**thyl acryl**am**ide).

Table S3. Comparison of cloud points for methacrylate-based polymers.

polymer	c / g·L^{-1}	pH	$T_{CP,523\ nm}$ / °C	Reference
PDEAEMA$_{109}$ [a]	1	6	70	4
	1	7	40	
PDMAEMA$_{108}$ [a]	1	7	76	5
	1	8	53	
	1	9	42.3	
	1	10	38.7	

[a] **p**oly(**die**thyl**a**mino**e**thyl **m**eth**a**crylate), [b] **p**oly(**dim**ethyl**a**mino**e**thyl **m**eth**a**crylate).

Temperature-dependent transmittance – switching the solubility behaviour with $K_3[Fe(CN)_6]$

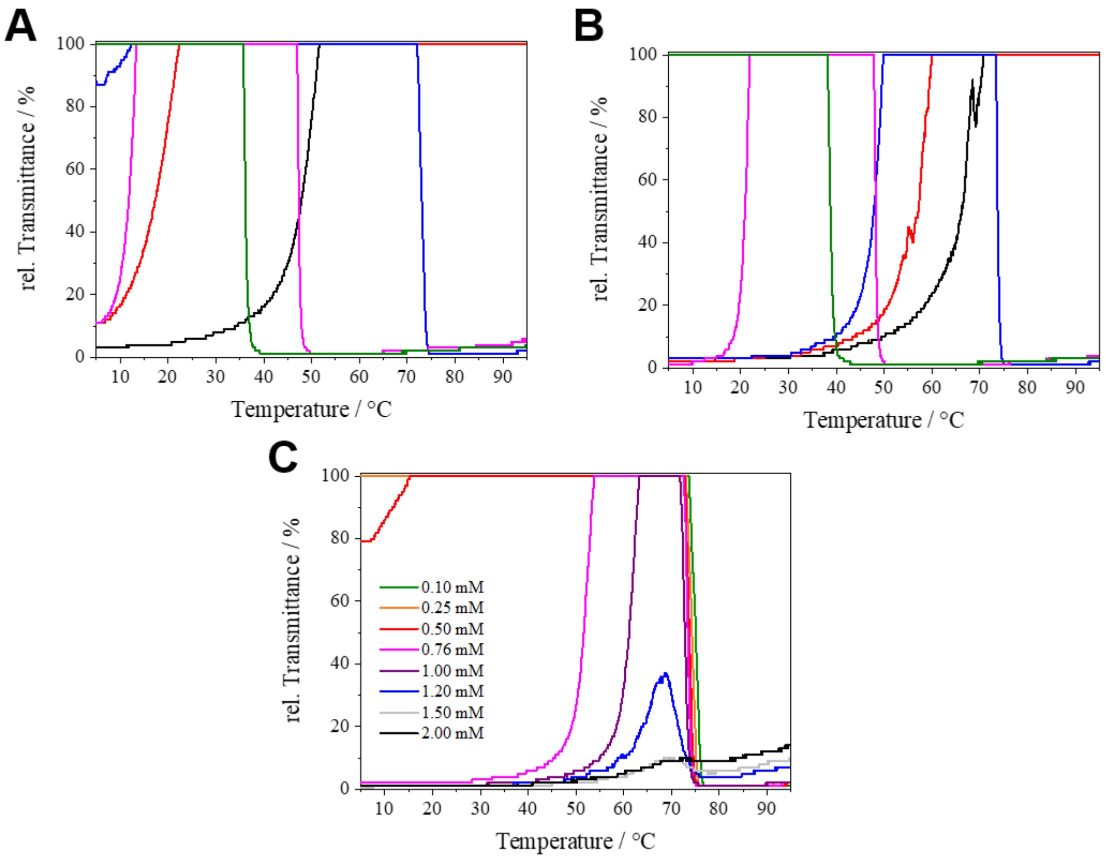

Figure S8. Temperature-dependent transmittance of PDEAEMAm$_{1030}$ (c = 1 g·L^{-1}) in the presence of $[Fe(CN)_6]^{3-}$ counterions in buffer solutions of different pH (pH = 6 (black), 7 (red), 8 (blue), 9 (magenta) and 10 (green)) with A) $c([Fe(CN)_6]^{3-})$ = 0.50 mM and B) $c([Fe(CN)_6]^{3-})$ = 0.75 mM. C) Dependence on $[Fe(CN)_6]^{3-}$ concentration in pH = 8 buffer solution.

Heterogeneous amidation

FT-IR spectra

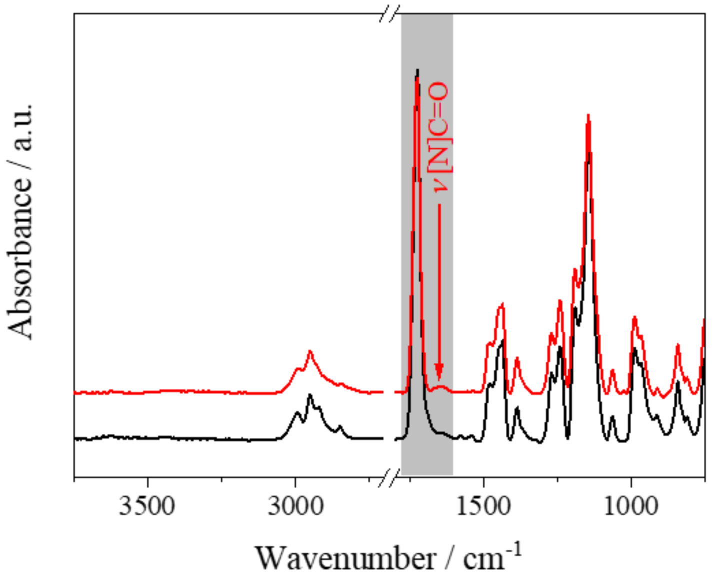

Figure S9. FT-IR spectra of a PMMA disc before (black) and after (red) heterogeneous amidation.

Contact angle

Table S4. Overview of the contact angle measurements before and after heterogeneous amidation.

sample	media	temperature / °C	contact angle / °
PMMA disc	deionized water	25	93 ± 2
	deionized water	55	83 ± 3
	pH 10 buffer	25	89 ± 4
	pH 10 buffer	55	75 ± 4
amidated PMMA disc	deionized water	25	49 ± 5
	pH 10 buffer	25	48 ± 6
	pH 10 buffer	55	77 ± 1

References

[1] P. A. Schaal, U. Simon, *Beilstein J. Nanotechnol.*, **2013**, *4*, 336–344.

[2] J. Schöbel, C. Hils, A. Weckwerth, M. Schlenk, C. Bojer, M. C. A. Stuart, J. Breu, S. Förster, A. Greiner, M. Karg, H. Schmalz. *Nanoscale* **2018**, *10*, 18257–18268.

[3] Z. Song, K. Wang, C. Gao, S. Wang, W. Zhang. *Macromolecules* **2016**, *49*, 162–171.

[4] A. Schmalz, M. Hanisch, H. Schmalz, A. H. E. Müller. *Polymer* **2010**, *51*, 1213–1217.

[5] F. A. Plamper, M. Ruppel, A. Schmalz, O. Borisov, M. Ballauff, A. H. E. Müller. *Macromolecules* **2007**, *40*, 8361–8366.

Author Contributions

Christian Hils: data curation, analysis and writing of original draft
Emma Fuchs and Franziska Eger: data curation and analysis
Dr. Judith Schöbel: writing of original draft, TOC
Dr. Holger Schmalz: project administration and writing of original draft
